# The Regulation of Pulmonary Vascular Tone by Neuropeptides and the Implications for Pulmonary Hypertension

**DOI:** 10.3389/fphys.2018.01167

**Published:** 2018-08-23

**Authors:** Charmaine C. W. Lo, Seyed M. Moosavi, Kristen J. Bubb

**Affiliations:** ^1^Kolling Institute of Medical Research, University of Sydney, St Leonards, NSW, Australia; ^2^School of Life Sciences, University of Technology Sydney, Ultimo, NSW, Australia

**Keywords:** substance P, calcitonin gene-related peptide, endothelial function, sensory C fibers, lung, right ventricle, pulmonary hypertension

## Abstract

Pulmonary hypertension (PH) is an incurable, chronic disease of small pulmonary vessels. Progressive remodeling of the pulmonary vasculature results in increased pulmonary vascular resistance (PVR). This causes secondary right heart failure. PVR is tightly regulated by a range of pulmonary vasodilators and constrictors. Endothelium-derived substances form the basis of most current PH treatments. This is particularly the case for pulmonary arterial hypertension. The major limitation of current treatments is their inability to reverse morphological changes. Thus, there is an unmet need for novel therapies to reduce the morbidity and mortality in PH. Microvessels in the lungs are highly innervated by sensory C fibers. Substance P and calcitonin gene-related peptide (CGRP) are released from C-fiber nerve endings. These neuropeptides can directly regulate vascular tone. Substance P tends to act as a vasoconstrictor in the pulmonary circulation and it increases in the lungs during experimental PH. The receptor for substance P, neurokinin 1 (NK1R), mediates increased pulmonary pressure. Deactivation of NK1R with antagonists, or depletion of substance P prevents PH development. CGRP is a potent pulmonary vasodilator. CGRP receptor antagonists cause elevated pulmonary pressure. Thus, the balance of these peptides is crucial within the pulmonary circulation (**Graphical Abstract**). Limited progress has been made in understanding their impact on pulmonary pathophysiology. This is an intriguing area of investigation to pursue. It may lead to promising new candidate therapies to combat this fatal disease. This review provides a summary of the current knowledge in this area. It also explores possible future directions for neuropeptides in PH.

## Introduction

Pulmonary hypertension (PH) is a rare chronic disease. Morphological changes in the pulmonary arterioles leads to increased pulmonary arterial pressure. Greater cardiac contractility occurs to overcome the higher pulmonary vascular resistance (PVR). The consequence is right heart remodeling, causing decompensating heart function (McLaughlin et al., 2015). The definition of PH is pulmonary arterial pressure >25 mmHg (or at least 30 mmHg upon exercise). Increased PVR and mean pulmonary wedge pressure occur. Patients suffering from PH show symptoms such as dyspnea, fatigue, chest pain, near syncope, syncope, leg or peripheral edema, angina, palpitations, and abdominal distension (Rich et al., 1987; Barst et al., 2004). PH is hard to diagnose as initial symptoms are non-specific. Fatigue, malaise, and exercise intolerance are often misdiagnosed as the patient being unfit. Asthma is also a common misdiagnosis due to the presence of dyspnoea. In fact, PH diagnosis often takes up to 2 years after symptom onset. Confirmation of PH sometimes occurs only after the progression of right heart failure.

**GRAPHICAL ABSTRACT:**
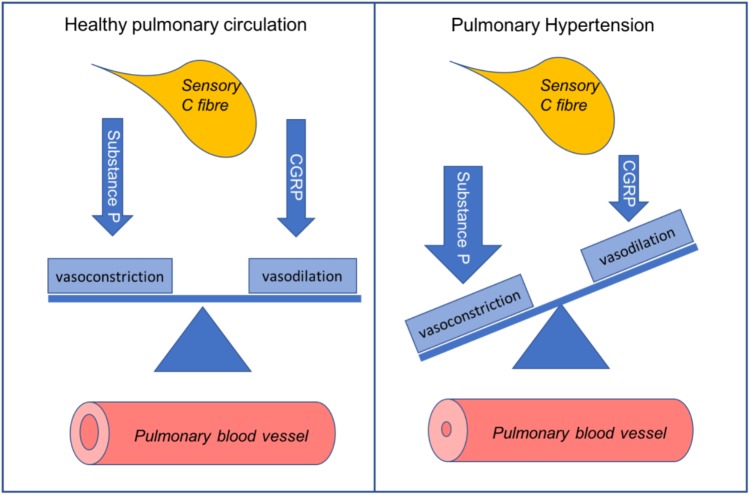


### Pulmonary Hypertension Disease Classifications

**FIGURE 1:**
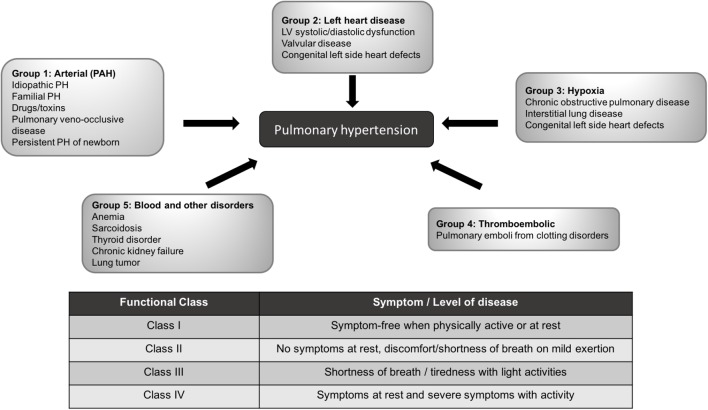
PH World Health Organization classification and functional class. Five different major sub-types of PH according to World Health Organization classification and functional classes are out-lined. Adapted from (Humbert and McLaughlin, 2009; Galie et al., 2016).

It is increasingly clear that PH has complex, multifactorial pathophysiology. In 1957 PH was categorized into five different groups based on the underlying cause of disease (Brenner, 1957), termed World Health Organization (WHO) groups. These have been further refined at subsequent World Symposiums on PH (Humbert and McLaughlin, 2009) (**Figure 1**).

**Group 1** PH is pulmonary arterial hypertension (PAH). The endothelium (inner) layer of blood vessels produces both vasodilators and vasoconstrictors. These are of extreme importance in regulating vascular tone. One of the defining features of PAH is the abnormal function of the pulmonary vascular endothelium. This results in an imbalance in dilators/constrictors, resulting in predominant vasoconstriction. PAH develops from many distinct contributing mechanisms. It is often idiopathic, occurring from (as yet) unknown cause. PAH may arise from hereditary factors, such as the inactivation of the *BMPR2* gene (Cogan et al., 2006; Morrell, 2006). It may develop following human immunodeficiency virus infection (Mehta et al., 2000). PAH can also be induced by drugs. For example, some anti-depressants (Garg et al., 2017) have been implicated in causing PAH and a spate of cases was caused by appetite suppressant drugs (Abenhaim et al., 1996) that have since been withdrawn from the market. **Group 2** PH is due to left-sided heart disease, either congenital or acquired. **Group 3** PH is due to pulmonary hypoxia, which occurs secondary to chronic lung diseases. **Group 4** PH is due to thromboembolic disorders and **Group 5** PH occurs from other disorders including anemia and other blood disorders, tumors, and chronic kidney failure.

For all PH subtypes, patients are also classified into functional classes (Galie et al., 2016), indicating disease severity (**Figure 1**). Patients tend to be diagnosed in functional class II-III and progressively worsen.

### The Unmet Need for Effective Treatments for Pulmonary Hypertension

Regardless of the cause of PH, there is common histopathology to all five groups. This includes hypertrophy of vascular smooth muscle cells, fibrosis, vascular wall remodeling and vessel obstruction. While the incidence of PH is low, at ∼15 cases per million people (Galie et al., 2015) the average survival time for a patient left untreated is only 2.8 years (Humbert et al., 2006; Peacock et al., 2007). Since the emergence of effective pharmaceuticals for PH, patient outcomes have substantially improved, as shown in REVEAL (Registry to Evaluate Early and Long-term PAH Disease Management) and French Consortium registries (D’Alonzo et al., 1991; McGoon et al., 2008; Frost et al., 2011; McGoon and Miller, 2012). Although there has been a substantial improvement in quality of life and longevity, the current treatments are far from ideal. For one thing, many patients become resistant to therapy (Morrell et al., 2009). In addition, PH remains a progressive and terminal disease. Current treatments have shown limited ability to reverse vascular and cardiac remodeling (Bubb et al., 2015). Thus, the search for novel and breakthrough treatments for PH continues in earnest.

In order to establish new PH treatments, it is important to determine how existing treatments can be improved. The first part of this review outlines the current treatments of PH. Subsequently, we review the effects of the sensory C-fiber-derived neuropeptides on the lung circulation. Several neuropeptides show promising effects and are being investigated for PH treatment. These include neuropeptides released from C-fibers: substance P and calcitonin gene-related peptide (CGRP). Both are important regulators of the pulmonary circulation. Modulation of either of the C-fiber-derived neuropeptides can reverse progression of experimental PH. Yet, their use has not progressed beyond pre-clinical research. We also discuss their potential as novel treatments for PH.

## Current Treatments For Pulmonary Hypertension

Current treatment options for all forms of PH include primary therapies directed at treating the underlying cause of the disease and broad therapies that alleviate the symptoms. General treatment prescribed at the discretion of the primary care physician includes the use of warfarin, diuretics and oxygen. The aims of these therapies are to alleviate volume and viscosity-induced pressure within the pulmonary circulation, reduce hypoxia, and treat right heart failure. Non-pharmaceutical approaches include lung transplant and double heart-lung transplant. Surgical approaches can be utilized in some cases, often when all other options have been exhausted. The primary pharmaceutical therapies that are approved for PH treatment are the main focus of this review and are detailed in subsequent sections.

### Targeting the Pulmonary Vascular Endothelium for Treatment of Pulmonary Arterial Hypertension

**FIGURE 2:**
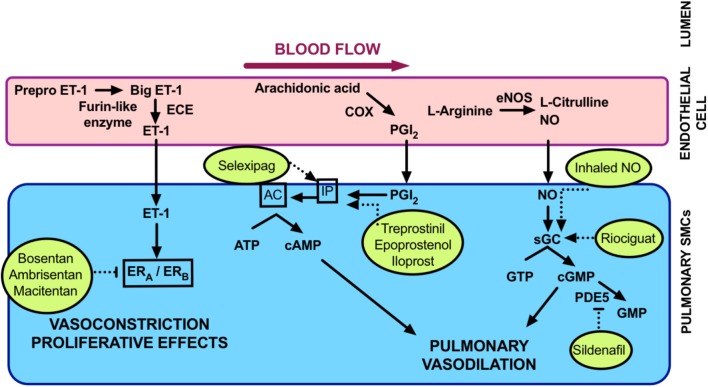
Summary of current PAH treatments. Currently licensed treatments for PAH are shown inside the circles with broken lines indicating their mode of action within vascular signaling pathways. AC, adenylate cyclase; cAMP, cyclic adenosine monophosphate; cGMP, cyclic guanosine monophosphate; COX, cyclooxygenase; ECE, endothelin converting enzyme; eNOS, endothelial nitric oxide synthase; ET-1, endothelin; ER_A,B_, endothelin receptor A,B; GTP, guanosine triphosphate; IP, inositol monophosphate; NO nitric oxide; PDE5, phosphodiesterase 5; PGI_2_, prostacyclin; sGC soluble guanylate cyclase.

For the purposes of this review we will focus mainly on Group 1 PAH. Vasoregulatory treatments are most successful in this type of PH (Austin and Loyd, 2015; Bubb et al., 2015; Prior et al., 2016). Thus, neuropeptides that we are investigating are most likely to impact PAH rather than other forms of PH. Disease-targeted treatment options for PAH are centered mainly around the control of pulmonary vascular tone. As outlined in **Figure 2**, major pathways that are currently targeted include endothelium-derived vasodilators and constrictors. The vasodilators of main interest are nitric oxide (NO) and prostacyclin. Both of these are susceptible to being decreased in bioavailability in PAH. These dilators are targeted at multiple levels. Treatments that either modulate their activity or inhibit their metabolism may be used. The endothelium-derived vasoconstrictor, endothelin (Galié et al., 2004) is the main culprit causing excessive vasoconstriction. Antagonists of the endothelin receptor/s are in clinical use for PAH. Calcium-channel blockers are also used to inhibit the calcium-dependent smooth muscle contraction.

#### Endothelium-Dependent Pulmonary Vasodilation

The reduction in pulmonary pressure by stimulating pulmonary endothelium-dependent vasodilation resulted in the first approved treatment for PAH. This continues to be the basis behind the development of the majority of PAH treatments.

##### Nitric oxide-cyclic guanosine monophosphate signaling

NO and the associated signaling pathways have a major role in promoting pulmonary vasodilation. The NO signaling pathway can be summarized as follows: NO activates soluble guanylate cyclase (sGC); this increases the second messenger, cyclic guanosine monophosphate (cGMP); cGMP then activates protein kinase G (PKG); PKG mediates vasodilation by decreasing intracellular calcium by a number of mechanisms. NO also mediates anti-inflammatory and anti-coagulatory effects. The low bioavailability of NO is a major occurrence in patients with PH. This is replicated in pre-clinical models of the disease (Michelakis, 2003). Upregulation of NO, either exogenously or endogenously, has been the primary aim of many decades of trials. Inhaled NO (Steiner et al., 2005) is useful in neonates suffering from persistent PH of the newborn. However, in adults it tends to result in methemoglobinemia, increased pulmonary oedema and a potentially life-threatening hypertensive rebound when therapy is ceased. It also suffers a short half-life and is prohibitively expensive and cumbersome for patients to administer (Michelakis, 2003; Steiner et al., 2005; Baliga et al., 2011). NO donors were developed in an attempt to improve the delivery of NO. Nebulised NONOates (Vanderford et al., 1994; Hampl et al., 1996) and inhaled glyceryl trinitrate (Goyal et al., 2006) effectively lower PVR. Yet they notoriously lack specificity for the pulmonary circulation (Baliga et al., 2011). Thus, targeting of the intermediates in the NO signaling pathway is a more attractive option for improving vasodilation.

In vascular endothelial cells, L-arginine is converted to NO (and the by-product L-citrulline) by endothelial NO synthase (eNOS) in a two-step oxidation reaction. This reaction utilizes nicotinamide adenine dinucleotide phosphate (NADPH), molecular oxygen and the essential cofactor, tetrahydrobiopterin (BH_4_) (Palmer et al., 1988; Andrew and Mayer, 1999; Forstermann, 2010). Phosphorylation of eNOS is an important regulatory mechanism for NO generation. It can also occur in response to shear stress, estrogen, vascular endothelial growth factor, and insulin. In normally functioning arteries the major stimulus for eNOS phosphorylation is increased intracellular calcium. This proceeds by binding of calcium to calmodulin in the oxygenase domain of eNOS, increasing the rate of NADPH electron transfer within the domains. In PAH (like other cardiovascular diseases) eNOS can exist in an ‘uncoupled state.’ Usually the substrate L-arginine or co-factor BH_4_ become rate-limiting. In this scenario, eNOS transfers electrons to molecular oxygen, rather than oxidizing L-arginine and superoxide is produced. Superoxide is a reactive oxygen species that is prevalent in vascular dysfunction (Verhaar et al., 2004). Supplementation with BH_4_ improves systemic hypertension (Landmesser et al., 2003). Evidence suggests that this strategy may also be effective in PAH (Baliga et al., 2011). Stimulating the coupling of eNOS and BH_4_ to promote NO rather than superoxide production has proven beneficial in experimental (Jin et al., 1992) and clinical PH (Saadjian et al., 1998). Increasing the substrate for NO by L-arginine administration can likewise attenuate PH in the experimental setting (Mitani et al., 1997) and acutely decreases PVR in PH patients (Mehta et al., 1995). Despite the extensive research, no therapeutic targets have been licensed that target NO generation.

Drugs that target the receptor for NO, sGC have proven to be more successful. Activation of sGC by NO is dependent on binding of NO to a heme prosthetic group. This stimulates a conformational change which activates the enzyme. Changes to the redox state of sGC, which can occur in PAH, can result in modulation of sGC expression or activity (Priviero and Webb, 2010; Baliga et al., 2011). Mice lacking sGC are sensitive to experimentally induced PH (Vermeersch et al., 2007). This makes sGC an important target in PH. Pharmacological stimulation of sGC is complicated and drugs fall into two distinct classes. sGC activators, (e.g., cinaciguat) are effective at activating sGC when it is in an oxidized or heme-free state. sGC stimulators (e.g., riociguat) stabilize the enzyme in its active configuration and upregulate sGC activity. There is an advantageous synergistic relationship of sGC stimulators and NO when they are co-administered (Stasch et al., 2011). Experimental PH was ameliorated after treatment with either sGC activators or stimulators (Dumitrascu et al., 2006; Stasch et al., 2011; Lang et al., 2012). Both classes of drugs are promising, but, sGC stimulators have progressed to the clinic first. The PATENT trials demonstrated improved clinical outcome for patients with PAH treated with riociguat (Ghofrani et al., 2012). Riociguat was first FDA-approved for treatment of PAH in 2013 and is now licensed in many countries. It is also the only drug licensed to treat group 4 PH after success in the CHEST trial (Lian et al., 2017).

The other major class of drugs to successfully treat PAH by targeting the NO-cGMP signaling pathway are phosphodiesterase (PDE) 5 inhibitors. PDEs metabolize cyclic nucleotides to their inactive form and some, including PDE5, are specific to cGMP. PDE5 is substantially upregulated in most forms of experimental and clinical PH (Murray et al., 2002; Sebkhi et al., 2003; Wharton et al., 2005; Schermuly et al., 2007) and this led to extensive testing of the efficacy of PDE5 inhibition for PH. Pre-clinical studies have provided insight into the therapeutic mechanisms of PDE5 inhibitors. It has been well-established that sildenafil is effective in inhibiting PH in experimentally induced models (Zhao et al., 2001; Schermuly et al., 2004; Steiner et al., 2005), and this is cGMP-dependent. Both sildenafil (Galie et al., 2005) and tadalafil (Galie et al., 2009) are effective in improving symptoms and outcomes of PH in patients.

##### Prostacyclin

Prostacyclin was first identified in 1976 (Moncada et al., 1976) and is the main eicosanoid produced by vascular endothelial cells. It is synthesized by cyclooxygenase-dependent conversion from arachidonic acid. Like NO, prostacyclin is also a classic endothelium-dependent vasodilator and also possesses anti-inflammatory and anti-coagulatory properties. Prostacyclin acts predominantly by binding with cell-surface IP receptors which stimulates adenylyl cyclase and cyclic adenosine monophosphate (cAMP) production with downstream effects mediated by protein kinase A (Mitchell et al., 2008). It is a particularly potent vasodilator in the lung circulation and PAH patients often exhibit decreased prostacyclin levels and/or reduced lung prostacyclin synthases (Mitchell et al., 2014). Prostacyclin analogs are a mainstay of PAH treatment but are generally restricted to functional class III and IV patients due mainly to limitations with their administration as outlined below. Prior to the use of epoprostenol (therapeutic prostacyclin) in 1995 (Barst et al., 1996), there was no therapy for PAH, and patients had a 1-year survival of 69% and a 5-year survival of 38% (D’Alonzo et al., 1991). When first introduced, epoprostenol was the only treatment available for PAH. It was widely used even though administration was challenging and side-effects were marked. It has been largely superseded by the next generation of therapeutic prostacyclins, iloprost and treprostinil. These are available in inhalers or by subcutaneous or intravenous routes. Recently the first non-prostacyclin that activates the cAMP signaling pathway has been developed. Selexipag is an IP receptor agonist, which has the benefit of being available in oral formulation (Mitchell et al., 2014). It has proven successful in Phase III trials (Sitbon et al., 2015) and has recently been licensed for PAH treatment.

#### Pulmonary Vasoconstriction by Endothelin

There are at least three isoforms of endothelin: endothelin (ET)-1, ET-2, and ET-3, of which ET-1 is the most potent and abundant in lungs (Matsumoto et al., 1989; Giaid et al., 1993a). First discovered in 1988 (Hickey et al., 1985; Yanagisawa et al., 1988), ET-1 is a peptide mainly produced by vascular endothelial cells. A number of physical factors affect ET-1 production, including shear stress, epinephrine, angiotensin II, growth factors, cytokines and free radicals (Marasciulo et al., 2006). ET-1 activity is mediated by ET receptors, ET_A_ and ET_B_, both expressed on vascular smooth muscle cells (Seo et al., 1994). ET-1 is produced by ET converting enzyme (ECE) from a precursor molecule of ET (La and Reid, 1995), big ET-1. ET-1 induces increased intracellular calcium via ET_A_ and ET_B_ receptor-mediated activation of phospholipase C (PLC) (Pollock et al., 1995) and increases mitogenesis by ET_B_-mediated activation of protein kinase C (Ohlstein et al., 1992), leading to both vasoconstriction and cell hyperplasia. ET_B_, which is also found on endothelial cells, stimulates local vasodilators such as NO and prostaglandins. It also plays a role in the clearance of ET-1, a feature unique to the lungs (Fukuroda et al., 1994; Kelland et al., 2010). PAH is associated with increased circulating ET-1 levels (Miyauchi et al., 1993). Expression of ET-1 has been found in plexiform lesions of lungs, where higher levels of ET-1 correlate with increased PVR and structural abnormalities (Giaid et al., 1993b; Bressollette et al., 2001). PAH patients may also have reduced clearance of ET-1 in the lung (Stewart et al., 1991), contributing to vasoconstriction.

Modulation of ET-1 has proven a successful strategy for treating PAH (Channick et al., 2004) and several endothelin receptor antagonists (ERA) are licensed for use. One of the first ERAs approved for PAH was bosentan, which antagonizes both ET_A_ and ET_B_ receptors. The clinical trials BREATH I and II (Rubin, 2002; Humbert et al., 2004) showed that non-selective oral bosentan delayed clinical worsening of PAH by improved exercise capacity, hemodynamics, and WHO functional class. Studies assessing longer term outcome found reduced mortality rates after bosentan use for PAH (McLaughlin et al., 2005; Sitbon et al., 2005). The EARLY trial found that bosentan slowed the deterioration of patients with group 2 PH. Ambrisentan is another ERA currently used for PAH, and is a selective antagonist of ET_A_ receptors (ET_A_:ET_B_ selectivity >4000:1), whereas bosentan (ET_A_:ET_B_ selectivity ∼300:1) is a non-selective antagonist (Elshaboury and Anderson, 2013). Ambrisentan improved mortality and survival times when tested in ARIES 1 and 2 randomized, double-blind, placebo-controlled, multi-centre efficacy studies (Galie et al., 2008). Macitentan is a dual ET_A_ and ET_B_ antagonist developed by modifying the structure of bosentan to increase safety and efficacy (Bolli et al., 2012). The phase III clinical trial (SERAPHIN), showed reduced morbidity and mortality, with improved cardiac hemodynamics and 6-min walk distance with macitentan use (Pulido et al., 2013). Macitentan is characterized by sustained receptor binding and has better tissue penetration (Iglarz et al., 2008; Gatfield et al., 2012). Common side effects of ERAs include peripheral oedema and flushing, nasopharyngitis, headache, and anemia. Bosentan is associated with teratogenesis and elevated transaminases in 10–12% of patients due to liver toxicity. Liver toxicity forced another ERA, sitaxentan, to be withdrawn from the market in 2010 due to hepatic necrosis (Odili and Abdullahi, 2014; Tran Thao et al., 2018). Ambrisentan (Galie et al., 2008) and macitentan appear superior in this respect, with lower risk of aminotransferase abnormalities.

## Investigation Of C-Fiber-Derived Neuropeptides As Novel Pulmonary Hypertension Therapies

Pulmonary vascular tone is controlled by many other factors, aside from those discussed above. There are a number of promising candidates for new PH treatment options, including the neuropeptides, substance P and CGRP. This pair of neuropeptides are of interest as both are released from sensory C-fibers. This complex has a well-characterized role in nociception and pain transduction, but this is not the focus of this review. Beyond their afferent role as nociceptors, sensory C-fibers have an efferent role. They can be stimulated in the periphery to release vasoactive neuropeptides. This leads to direct and immediate modulation of vascular tone (Holzer, 1991). The endogenous stimulation of C-fibers largely involves inflammatory signals. C-fibers are pharmacologically characterized by their sensitivity to noxious stimuli such as heat, acidic pH and capsaicin, the ‘hot’ component in chili peppers.

### Use of Capsaicin in Isolating Neuropeptide Activity

**FIGURE 3:**
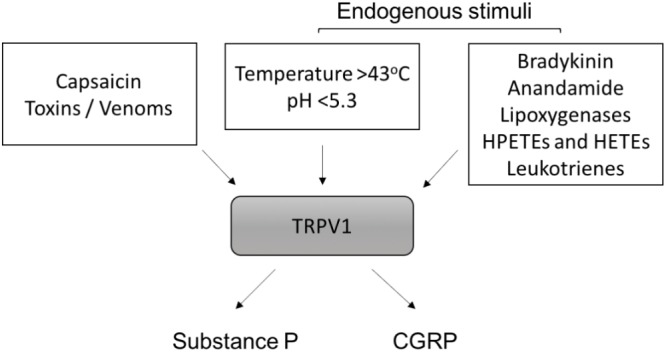
Activation of TRPV1 and neuropeptide release. Exogenous and endogenous stimuli that activate TRPV1 are shown. HPETEs (hydroperoxyeicosatetraenoic) and HETEs (hydroxyeicosatetraenoic) refer to eicosanoid derivatives of arachidonic acid. Once activated, TRPV1 on sensory C-fibers releases substance P and calcitonin-gene related peptide (CGRP) from nerve endings.

Capsaicin has proven to be a useful pharmacological tool for investigating the actions of the C-fiber-derived neuropeptides (**Figure 3**). Acutely, capsaicin can activate C-fibers to release substance P and CGRP. Yet, with prolonged use it leads to depletion of nerve endings of their neuropeptide content via retrograde transport of nerve growth factor (NGF) to the cell bodies of sensory nerves (Burks et al., 1985; Juranek and Lembeck, 1997). As an example of this, in rats, capsaicin can permanently degenerate C-fiber afferents leading to reductions of neuropeptide levels. This is a promising strategy for alleviating chronic pain (Lynn, 1990). Capsaicin has been used extensively to characterize the contributions of neuropeptides to vascular regulation. The role of CGRP and substance P in the cardiovascular system, are discussed in detail in the following sections.

### Calcitonin-Gene Related Peptide (CGRP)

First identified in 1982, CGRP is a polypeptide which has 37- amino acids (Amara et al., 1982; Rosenfeld et al., 1983; Wimalawansa, 1996) and belongs to a larger peptide family including calcitonin. CGRP is expressed in neuronal tissue and in central, peripheral and enteric nervous systems (Mulderry et al., 1985b). It can be found in both unmyelinated sensory C fibers and myelinated Aδ fiber. Immunocytochemistry studies showed cells within the central nervous system (CNS), peripheral nerves in the heart, airway mucosa and general vasculature all have high amounts of CGRP (Rosenfeld et al., 1983; Mulderry et al., 1985c; Tjen et al., 1998). It was originally reported that CGRP could mediate sympathetic efflux from brain (Fisher et al., 1983), and that intravenous delivery could lower blood pressure and increase heart rate (Fisher et al., 1983; Marshall et al., 1986). It was then crucially discovered that CGRP has direct vasodilator activity (Brain et al., 1985). Perivascular nerves are the major source of plasma CGRP and deficiency of perivascular nerves has been shown in certain types of hypertension (Wimalawansa, 1996; Smillie and Brain, 2011). CGRP is widely believed to have anti-inflammatory effects (Gomes et al., 2005). Inflammation-mediated nerve damage can upregulate CGRP synthesis (Amara et al., 1982; Rosenfeld et al., 1984; Donnerer and Stein, 1992). In vasculature, terminal CGRPergic sensory fibers are located in all layers of the vascular smooth muscle (Rosenfeld et al., 1983) (Amara et al., 1985; Mulderry et al., 1985a). CGRP can be found in perivascular neurons common to all vascular beds, where expressions are higher in arterial than venous tissues in human coronary arteries (Amara et al., 1985). Receptors for CGRP have also been found in both the media and intima of resistance vessels and in endothelial cells (Mulderry et al., 1985a; Hagner et al., 2002). The wide distribution of CGRP and its receptor in the vasculature makes CGRP a promising future drug target.

#### CGRP Is a Potent Vasodilator

Calcitonin gene-related peptide is a potent vasodilator in the systemic circulation and other localized vascular beds. (Brain et al., 1985; Kubota et al., 1985; Greenberg et al., 1987; van Rossum et al., 1997). CGRP achieves vasodilation by receptor-mediated activation of adenylyl cyclase and cAMP (Aiyar et al., 1997), and may also have some cross-talk with NO (Chen and Guth, 1995). Patients with hypertension have increased plasma CGRP concentrations (Masuda et al., 1992; Li et al., 2009). Circulating CGRP levels correlate with systolic and diastolic pressures in severe hypertension (Edvinsson et al., 1992). Dose-dependent increases in plasma CGRP levels were found when blood pressure was artificially raised with angiotensin II in normotensive volunteers (Portaluppi et al., 1993). These studies suggest that raised plasma CGRP is a compensatory mechanism to overcome high blood pressure. While CGRP is the most potent neuropeptide vasodilator, it suffers from a short half-life that may limit its clinical use. To overcome this a long lasting acylated α-CGRP analog (half-life ≥ 7 h) was used in a pre-clinical study. This effectively alleviated hypertension (Aubdool et al., 2017), further supporting the notion of CGRP acting as an endogenous anti-hypertensive agent.

#### CGRP in Pulmonary Hypertension

Similar to systemic circulation, CGRP is also a major vasodilator in pulmonary arteries (McCormack et al., 1989a). Using experimental models, it appears that upregulating CGRP signaling protects against increased PVR in PAH (**Table 1**). Pulmonary vascular remodeling, right ventricular systolic pressure and right ventricular hypertrophy can all be ameliorated by the infusion of CGRP in experimental PH (Keith and Ekman, 1992; Tjen et al., 1992). Decreased CGRP activity, by CGRP receptor antagonism resulted in exacerbated PH in rats (Tjen et al., 1992). More interestingly, gene therapy with CGRP has also proven to be effective in preventing PH characteristics *in vivo* and *in vitro* in experimental PH models (Champion et al., 2000; Bivalacqua et al., 2002; Chattergoon et al., 2005; Zhao et al., 2007). Endothelial progenitor cells that were transfected with CGRP and directed to the pulmonary vasculature of rats led to decreased PVR (Zhao et al., 2007). This suggests that PH-associated endothelial dysfunction may be corrected by CGRP.

**Table 1:** Role of neuropeptides in experimental models of PH.

Species	PH model	Treatment	Effect	Reference
**TRPV1**				
Rat	Monocrotaline	Capsaicin	↓ PH, ↓RVH	Zhou and Lai, 1993; Katzman and Lai, 2000
Rat	Pulmonary banding	Capsaicin	↓ PH, ↓RVH	Xu et al., 2017
Rat	Hypoxia	Capsaicin	↑ PH, ↑RVH	Tjen et al., 1998
Rat	Perinatal hypoxia/ monocrotaline	Capsaicin	↓ PAP	Chen et al., 2012
**CGRP**				
Rat	Post-natal hypoxia		↓ CGRP ↑ PH	Keith et al., 2000
Mouse	Hypoxia	CGRP gene transfer	↓ PH, ↓RVH	Champion et al., 2000; Bivalacqua et al., 2002
Rat	Hypoxia	CGRP	↓ PH, ↓RVH	Tjen et al., 1992
Rat	Hypoxia	CGRP receptor impaired	↑ PH	Qing and Keith, 2003
Rat	Hypoxia	CGRP infusion	↓ PH, ↓RVH	Qing and Keith, 2003
**Substance P**				
Rat	Perinatal hypoxia/ monocrotaline		↑ Substance P	Chen et al., 2012
Rat	Monocrotaline		↑ Substance P	Zhou and Lai, 1993
Rat	Hypoxia	NK1R antagonist	↓ PAP	Chen et al., 1999
Rat	Hypoxia	NK1R activation	↑ PAP	Chen et al., 1999


*CGRP, calcitonin gene-related peptide; NK1R, neurokinin 1 receptor; PH, pulmonary hypertension; RVH, right ventricular hypertrophy.*

Interestingly, patients with higher CGRP levels had more severe PAH (Bartosik et al., 2002; Zhang et al., 2006). Likewise, chronic hypoxia-induced PH in rats was associated with higher levels of CGRP. The latter correlated with increased arterial medial hypertrophy and right ventricular systolic pressure and hypertrophy (Keith and Ekman, 1992). These studies hint at a role for CGRP in the pathophysiology of PAH. Taken in context with the therapeutic potential of CGRP for lowering pulmonary pressure, it is likely that increased CGRP levels reflect a compensatory upregulation to overcome the high pulmonary pressure. If this is the case, CGRP may be a useful clinical biomarker for PAH diagnosis.

### Substance P

Substance P was first discovered in 1931 after tissue extract containing a previously unknown compound was demonstrated to stimulate rabbit intestinal contraction (V Euler and Gaddum, 1931). Several decades later the structure of substance P, as a polypeptide containing 11 amino acids, was resolved (Chang et al., 1971). It is now well established that substance P is involved in many physiological and pathological effects, mediating touch, pain and temperature (Lembeck and Holzer, 1979; Holzer, 1988). While substance P is mostly known for its role in pain and neurogenic inflammation and is found in the central nervous system (Mantyh, 2002), it also mediates effects via receptors in non-neuronal tissues. Substance P modulates cell proliferation and cytokine production, mediates interaction between immune cells and neurons (de Avila et al., 2014; Mashaghi, 2016) and is involved in immunoregulation (Payan and Goetzl, 1985; Stead et al., 1987, 1989; Haines et al., 1993; Ho et al., 1997; Schratzberger et al., 1997). The main receptor for substance P is neurokinin 1 receptor (NK1R), which belongs to the tachykinin receptor family of G protein-coupled receptors. When substance P binds to NK1R a scaffold complex can be formed, resulting in endocytosis and stimulation of intracellular signaling via mitogen activated protein kinases (Grady et al., 1995; DeFea et al., 2000).

#### Substance P Is an Indiscriminate Regulator of Vascular Tone

**FIGURE 4:**
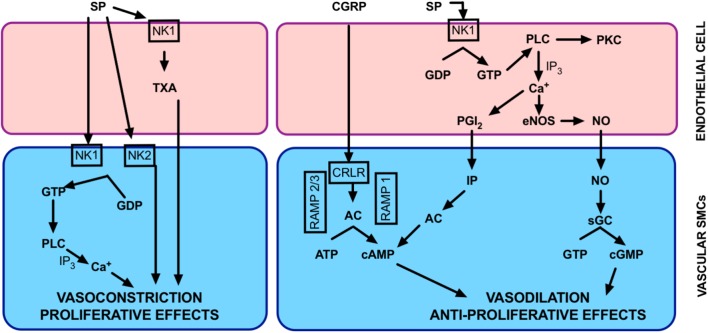
Overview of neuropeptide function in the lung circulation. Proposed mechanisms by which CGRP and Substance P may be effective in regulating pulmonary vascular tone. AC, adenylate cyclase; cAMP, cyclic adenosine monophosphate; cGMP, cyclic guanosine monophosphate; CRLR, calcitonin receptor-like receptor; eNOS, endothelial nitric oxide synthase; GDP, guanosine diphosphate; GTP, guanosine triphosphate; IP_3_, inositol triphosphate; MAPK, mitogen activated protein kinase; NK, neurokinin; NO nitric oxide; PGI_2_, prostacyclin; PKC, protein kinase C, PLC, phospholipase C; RAMP-1, receptor activity-modifying protein-1; sGC, soluble guanylate cyclase; TXA, thromboxane.

In the vasculature, substance P promotes proliferation of smooth muscle cells (Payan, 1985). It also influences vascular tone in a complex manner. It has a dual role of being a vasoconstrictor or vasodilator, depending on the circumstances and the type of vessel it stimulates (Worthen et al., 1985). The vascular response to substance P is largely mediated by NK1R activation. Substance P has a higher affinity for NK1R compared to NK2R and NK3R. There is little evidence to suggest a role for the other isoforms in vascular reactivity (Maggi et al., 1990; Constantine et al., 1991; Hall and Brain, 1994; Berthiaume et al., 1995; Corboz et al., 1998). Whether the effect of Substance P is to cause vasodilation or vasoconstriction is dependent on where NK1R is located. Activation of NK1R on smooth muscle cells may induce vasoconstriction. If NK1R on endothelial cells are stimulated, calcium-induced activation of the vascular endothelium can occur. This results in production of endothelium-derived vasodilators. Yet, endothelial NK1R activation can also result in thromboxane-mediated vasoconstriction (**Figure 4**). Interestingly, global knockout of the NK1R gene (*Tacr1*) resulted in no alteration to vascular function in isolated systemic arteries (Moyes et al., 2016). Given that both smooth muscle and endothelial cells were lacking NK1R in these mice, it may explain the phenotype. Vascular function would be best explored using tissue-specific knockout mice (i.e., smooth muscle and/or endothelial cell knockout) to truly determine functional relevance of NK1R.

##### Substance P as a vasodilator

Substance P can be a potent vasodilator of some vessel types and this has been shown in isolated vessels from both humans (Bodelsson and Stjernquist, 1992; Onoue et al., 1994; Wallerstedt and Bodelsson, 1997) and animals (D’Orleans-Juste et al., 1985; Bolton and Clapp, 1986; McCormack et al., 1989b; Toda et al., 1991) and *in vivo* (McEwan et al., 1988; Beattie et al., 1993; Hall and Brain, 1994; Strobel et al., 1996). Vasodilation induced by substance P via NK1R can occur through both endothelium-dependent (D’Orleans-Juste et al., 1985; Bolton and Clapp, 1986; Onoue et al., 1994) and -independent mechanisms (Enokibori et al., 1994). Endothelium-dependent vasodilation (**Figure 4**) after substance P stimulation can be via any of the major endothelium-dependent pathways; NO (Rosenblum et al., 1993; Holton et al., 2010), prostacyclin (Bodelsson and Stjernquist, 1994) or endothelium-dependent hyperpolarization of the smooth muscle membranes (Petersson et al., 1995; Wallerstedt and Bodelsson, 1997). This can also occur through NK1R in isolated pulmonary vessels (Corboz et al., 1998; Pedersen et al., 2000). Substance P-induced pulmonary vasodilation tends to occur at low concentrations (McCormack et al., 1989b) and can be transient in nature (Maxwell, 1968).

##### Substance P as a vasoconstrictor

Whilst it is often considered a systemic vasodilator, substance P can also induce a myogenic ‘response,’ or pressure-induced constriction (Scotland et al., 2004). When substance P release is stimulated, it can induce coronary vasoconstriction and increased blood pressure (Bubb et al., 2013) and it may have a role in the pathogenesis of hypertension (Faulhaber et al., 1987; Kohlmann et al., 1997). Activation of the NK1R on smooth muscle cells results in activation of phospholipase C. This creates a transient increase of inositol 1,4,5 triphosphate (Takeda et al., 1991) and increased intracellular calcium (Krause et al., 1992), resulting in vasoconstriction. Substance P primarily causes vasoconstriction in pulmonary arteries (Selig et al., 1988; McCormack et al., 1989b; Shirahase et al., 1995), which can be mediated via NK1R via thromboxane or NK2R (**Figure 4**). This appears to be largely pulmonary-specific (Selig et al., 1988) and likely involves complex receptor interactions. At higher substance P concentrations, pulmonary vasoconstriction dominates over vasodilation (McCormack et al., 1989b).

#### Substance P in Pulmonary Hypertension

The complexities around the dual vasodilator/vasoconstrictor role of substance P in the pulmonary circulation (**Figure 4**) make it difficult to predict what role it has (if any) in PH. Infusion of substance P has minimal vasodilating effect on patients with PAH (Uren et al., 1992; Brett et al., 1996; Cailes et al., 1998). Thus, is has been postulated that substance P dysfunction is an underlying cause of PAH. On the other hand, the dominant role of substance P as a pulmonary–specific vasoconstrictor led to the hypothesis that substance P overactivity is causative in PAH. Pre-clinical models of PH are associated with increased lung substance P (Zhou and Lai, 1993; Chen et al., 2012). Pulmonary pressure can be decreased by depleting substance P (**Table 1**) or by using NK1R antagonists (Zhou and Lai, 1993; Chen et al., 1999, 2012). Likewise, activation of NK1R can induce increased pulmonary pressure (Chen et al., 1999). Substance P is involved in lung vascular remodeling, possibly due to increased oxidative stress (Springer and Fischer, 2003). Increased PVR has also been attributed to inflammatory stimulation of mitogen-activated protein kinase pathway by substance P. This is alleviated by capsaicin-induced depletion (Xu et al., 2017). In model systems using hypoxia to simulate PH, substance P release is increased (Lindefors et al., 1986). Based upon this body of literature it would appear that substance P is indeed a promising candidate for reducing PVR.

Further research should be conducted to pursue the intricate mechanisms of substance P in PAH. As NK1R antagonists can lower pulmonary pressure in rats, they may be the best pharmacological target to consider for humans. NK1R antagonists are in clinical use as anti-emetics. They are usually prescribed to take prior to chemotherapy or surgery. Aprepitant is available as an oral formulation and is well tolerated (Martinez and Philipp, 2016). It has been investigated for use as an anti-inflammatory agent. Interestingly, reported side-effects include low blood pressure. If subsequent pre-clinical studies are positive, a clinical trial in PH could conceivably proceed using this safety-approved formulation.

### Transient Receptor Potential Vanilloid Type 1 (TRPV1) in Vascular Regulation

There are a number of signaling intermediates in C-fiber-derived neuropeptide release. One of the best characterized in the vasculature is TRPV1. TRPV1 activation results in substance P and/or CGRP release from peripheral nerve terminals (Holzer, 1991; Gibbons et al., 2010). TRPV1 receptors are non-selective cation channels located predominantly on sensory nerve endings (Tominaga and Julius, 2000) but they also reside on cells of many peripheral tissue types. They are present in the entire respiratory tract (Zholos, 2015) and located on both smooth muscle and endothelial cells of the vasculature. TRPV1 receptors are nociceptors that are highly responsive to pro-inflammatory stimuli and are activated by a range of endogenous and exogenous factors (**Figure 3**). These include temperature, pH, bradykinin, anandamide, arachidonic acid metabolites such as 20-hydroxyeicosatetraenoic acid (Wen et al., 2012), spider toxins, and most famously, capsaicin, (Caterina et al., 1997; Devesa et al., 2011). TRPV1 receptors have a binding site for capsaicin. The complicated mechanism for activation is still being unraveled and great progress has been made since 2013 when breakthrough structural information was first reported (Yang and Zheng, 2017). TRPV1 activation is involved in a variety of cardiovascular pathologies, including the modulation of atherosclerosis (Li et al., 2014; Xiong et al., 2016), myogenic tone (Scotland et al., 2004; Bubb et al., 2013), systemic arterial pressure (Bubb et al., 2013) and hypertension (Hao et al., 2011), congestive heart failure (Gao et al., 2014; Lang et al., 2015), vascular remodeling (Chen et al., 2010), haemorrhagic shock (Akabori et al., 2007) and sepsis (Chen et al., 2018).

#### Evidence of a Role for TRPV1 in Pulmonary Hypertension

Pulmonary hypertension is characterized by pulmonary smooth muscle cell hyperproliferation causing remodeling of the smooth muscle cell layer and impacting on PVR. Although it is likely that TRPV1 activation in sensory nerves is the key to modulation of pulmonary circulation, it is possible that some direct effects in smooth muscle cells could contribute to the PH phenotype. Activation of TRPV1 on cultured pulmonary smooth muscle cells (SMC) results in enhanced proliferation (Randhawa and Jaggi, 2017), increased intracellular calcium and stimulates cell migration (Martin et al., 2012). Under laboratory conditions, PH can be simulated by exposing cells to chronic hypoxia. Using this model, TRPV1 expression can increase (Wang et al., 2008) with corresponding increased intracellular calcium and reorganization of cytoskeletal architecture. TRPV1 blockade with capsazepine can abolish these effects (Parpaite et al., 2016). There are a number of studies assessing the role of TRPV1 by using capsaicin to desensitize it (**Table 1**). These studies are further evidence of the importance of TRPV1 in PH. The contribution of individual neuropeptides downstream of TRPV1 activation is complex and has been discussed in the relevant sections of this review.

## Direct Cardiac Effects Of Sensory C-Fiber Neuropeptides

**FIGURE 5:**
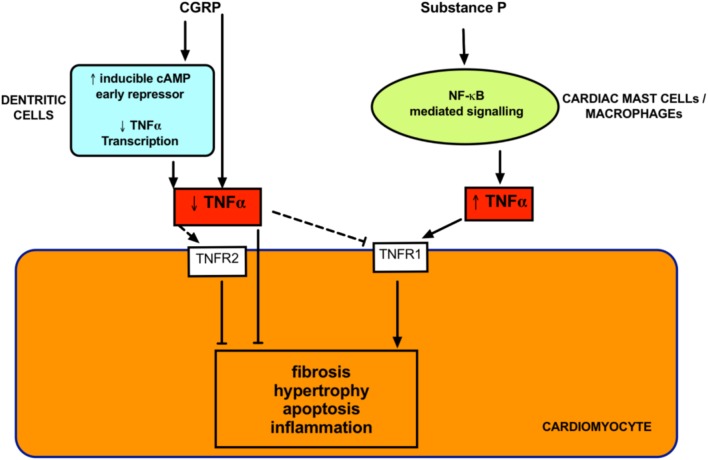
Proposed direct cardiac effects of neuropeptides. Both Substance P and CGRP modulate tumor necrosis factor α (TNFα) signaling. TNF-α is crucial in mediating cardiac remodeling and these effects can occur via activation of receptors (TNFR1 and TNFR2) on cardiomyocytes. Substance P promotes cardiac remodeling via TNFα release from inflammatory cells. CGRP inhibits TNFα and decreases remodeling in diseased models. Dashed lines indicates unknown but postulated mechanisms.

The increased PVR in all groups of PH generally leads to right ventricular cardiac remodeling due to the increased right ventricular afterload. This remodeling eventually leads to right heart failure and accounts for mortality in most patients. The right heart remodeling is the most difficult component to treat. Similar to left-sided heart failure, there are a paucity of drugs that are effective in reversing cardiac remodeling. Many PH patients remain undiagnosed until right heart remodeling is already established. Therefore, this is a significant clinical problem. It is important to investigate the direct cardiac effects of potential therapies for PH. Even if the effect on vascular resistance is modest, any direct effects on cardiac remodeling would increase the usefulness of the therapy. Current treatments tend to be disappointing in this respect (Michelakis, 2003; Steiner et al., 2005; Baliga et al., 2011). In PAH, where the treatments cause a significant decrease in right heart afterload, the impact on the right ventricular mortality is minimal (Westerhof et al., 2017). Promisingly, both CGRP and substance P have been reported to have direct cardiac effects (Ejaz et al., 2011) as outlined below (**Figure 5**).

### CGRP in Heart Failure and Cardiac Remodeling

Calcitonin gene-related peptide receptors are located throughout the myocardium (Ieda et al., 2006). There is evidence of densely located CGRP-containing nerve fibers around the coronary arteries, papillary muscles, sinoatrial and atrioventricular nodes (Mulderry et al., 1985b). CGRP protects against cardiac hypertrophy in a pre-clinical model of heart failure (Li et al., 2010). CGRP levels can be increased in heart failure (Hsu et al., 2005). Whether this is causative or compensatory is unknown. However, there is evidence to support the latter notion. Infusion of CGRP increases myocardial contractility (Gennari et al., 1990) and improves circulation in coronary disease (Gennari et al., 1990; Dubois-Rande et al., 1992; Stevenson et al., 1992). On a cellular level, CGRP inhibits generation of pro-inflammatory tumor necrosis factor (TNF)-α and prevents interstitial and perivascular fibrosis (Li et al., 2013). CGRP also ameliorates cardiomyocyte apoptosis via the Bcl-2/Bax pathway (Ma et al., 2013) and has direct anti-proliferative effects on cardiac fibroblasts (Li et al., 2016). CGRP has positive cardiac ionotropic effects that are mostly abolished by both a CGRP antagonist and a PI3 kinase inhibitor (Al-Rubaiee et al., 2013). A CGRP analog alleviated pre-clinical diabetes-induced heart failure and remodeling due to reduced α-smooth muscle actin and transforming growth factor (TGF)-β_1_ (Aubdool et al., 2017). Therefore, CGRP appears to be cardioprotective. Even more promising for PH, is the recent finding that rutacarpine, which stimulates CGRP release, was able to reverse right heart remodeling in experimental PH and this was attributed to p27-dependent signaling (Li et al., 2016).

### Substance P in Heart Failure and Cardiac Remodeling

In the human heart, substance P has been found in intrinsic cardiac ganglia (Wharton et al., 1990; Hoover et al., 2009), coronary vessels (Laine et al., 2000), and within myocardium (Weihe et al., 1981; Rechardt et al., 1986). Substance P appears to have a damaging role in heart disease (Dehlin and Levick, 2014), hinting at a direct effect of substance P on myocardial tissue. Pro-substance P (a stable surrogate for substance P) is an independent predictor of recurrent acute myocardial infarction, heart failure and cardiac mortality (Ng et al., 2014). Levels of substance P are increased in congestive heart failure (Valdemarsson et al., 1991). Substance P promotes hypertrophy in isolated cardiomyocytes, suggesting this effect is direct and independent of systemic factors (Church et al., 1996). Several pre-clinical models have demonstrated a role for substance P in mediating heart failure. Cardiac hypertrophy, apoptosis and dilated cardiomyopathy were absent from substance P knockout mice (D’Souza et al., 2007). Genetic deletion of *TAC1*, which encodes substance P was protective against increased cardiac mast cell density and TNFα upregulation and associated cardiac remodeling (Melendez et al., 2011). Inhibition of NK1R also improved cardiac function and ameliorated cardiac hypertrophy in models of heart failure (Melendez et al., 2011; Robinson et al., 2015). Substance P appears to be a mediator of cardiac toxicity induced by doxorubicin, which is a chemotherapy agent that causes cardiomyocyte death. Inhibition of substance P resulted in lower cardiomyocyte apoptosis after doxorubicin treatment to an isolated cell line (Robinson et al., 2016). Taken together, these findings are clinically exciting, as it suggests that substance P-antagonist based therapies may be able to directly improve right ventricular parameters above simply relieving right ventricular afterload for PAH treatment.

## What Is Not Known? Future Prospects For Neuropeptides As A Treatment Strategy For Pulmonary Arterial Hypertension

From a relatively small pool of research we can conclude that the neuropeptides, substance P and CGRP, have the ability to regulate both systemic and pulmonary arterial pressure. We have also provided evidence that these neuropeptides regulate vascular smooth and cardiac muscle cell remodeling under diseased conditions. However, there is not yet a comprehensive answer on whether these neuropeptides are strong signaling targets for treating PAH. CGRP is a proven potent dilator of systemic and pulmonary vasculature and appears to have favorable effects on cardiac remodeling. Based on what we know, stimulation of endogenous CGRP signaling or exogenous administration of stable analogs should be good candidates for the treatment of PAH. Yet only a handful of studies have investigated this and it has not gone further than pre-clinical investigation. The next step would be to thoroughly characterize the potential for CGRP in reversing all aspects of PAH. It is important to show this in three or more different PH models, before embarking on a Phase II trial.

The clinical appeal of substance P is more distant as there are still inconsistencies in identifying the precise vascular regulatory function of this neuropeptide. From the pulmonary literature, it appears that the over-arching role of substance P is likely to be as a vasoconstrictor. This is contrary to its effect in the systemic circulation, where it seems to predominantly cause vasodilation. If this is proven to be the case, it is a very attractive property for PAH treatment. A major adverse effect for some PAH therapies has been the unacceptable lowering of systemic arterial pressure. This is an important factor to consider for PAH treatment, especially in regard to using combination therapies.

Combination therapies are emerging as a promising treatment regimen for PAH, while we wait for development of novel compounds. An effective PVR-lowering combination has involved upregulation of cGMP generation with concurrent inhibition of cGMP metabolism. However, this is not always possible. For example, the use of sildenafil and long-acting nitrates can cause life-threatening hypotension (Cheitlin et al., 1999). Using pre-clinical models, an alternative strategy has emerged. Stimulation of a different ‘pool’ of cGMP, derived from particulate guanylate cyclase activity rather than sGC (Baliga et al., 2008), and combining this with sildenafil, has been a success. This has resulted in a far more pulmonary-specific vasodilation, negating the hypotensive effects. A similar approach could apply for substance P. Antagonism of NK1R would presumably block the pulmonary vasoconstriction of substance P, but also prevent excessive systemic dilation to some degree. A hypothetical treatment strategy could involve using NK1R antagonists alongside already approved PAH therapies such as PDE5 inhibition or sGC stimulation. This may result in a pulmonary-specific dilation, as substance P-induced systemic vasodilation would be prevented at the same time as pulmonary vasoconstriction is lowered. Pre-clinical studies should focus on investigating the substance P axis alongside current treatments, or in combination with other promising candidates. This could identify new strategies that are truly able to target multiple etiologies of PAH and are an essential starting point. A similar strategy should be utilized when investigating CGRP for PAH therapy. It is likely that CGRP-based pharmaceuticals may have significant and unacceptable blood pressure lowering effects, similar to the organic nitrates. Therefore dose-selection and combination approaches would be essential in establishing CGRP as a therapy.

As TRPV1 activation results in release of both neuropeptides, it may also make an interesting target. If antagonism of TRPV1 lowers substance P, this may be beneficial for PAH. It would also presumably lower CGRP, which may not be beneficial. It probably depends on the release pattern of CGRP/substance P in PH lungs and little is known about that. The majority of studies so far have found that depleting the C-fiber nerve endings by using capsaicin has generally resulted in improvements in PH models (**Table 1**). This suggests that inhibiting sensory C-fibers predominantly impairs substance P-induced pulmonary constriction without a major influence on CGRP-induced dilation. This may be reflective of the mechanisms at play in regards to the role of TRPV1 in PAH. Future research should focus on whether complete inhibition of TRPV1 could be effective as a PH therapy, or whether the balance could be shifted more toward CGRP-production with inhibition of substance P. Of course, with any strategy, consideration must be given to the consequences for other organ systems. Complete inhibition of either TRPV1 or substance P may elicit unexpected effects and this should be thoroughly investigated in PH models. Use of targeted therapy to the pulmonary vasculature and/or heart would help to elucidate the role of both neuropeptides. Also, receptor-specific therapy could be considered. One study has investigated all three neurokinin receptors, but it indicated no role of NK2R and NK3R (Corboz et al., 1998). The little that has been done suggests that NK1R is likely to be the primary mediator of pulmonary vasoconstriction, but the other neurokinins should not yet be ruled out.

Another interesting area to explore could be related to neutral endopeptidases (NEP), which are the most important enzymes in the degradation of tachykinins, including substance P (Skidgel et al., 1984). Interestingly, there is a decent body of evidence to suggest that NEP inhibitors could be a promising PAH therapy, and they are currently in clinical trial. In addition to tachykinins, NEPs also degrade a selection of other peptides, including pulmonary vasodilating natriuretic peptides and pulmonary vasoconstrictors such as ET-1 (Abassi et al., 1992). NEPs appear to have an underlying role in the pathogenesis of PH (Dempsey et al., 2009). NEP inhibitors have produced promising results in models of PH, both as monotherapy (Klinger et al., 1993; Thompson et al., 2012) or in combination with PDE5 inhibition (Baliga et al., 2008). This is largely due to their ability to increase circulating atrial natriuretic peptide. However, in addition to increasing pulmonary vasodilators, NEP inhibitors may result in increased circulating substance P (in addition to other vasoconstrictors such as ET-1 and angiotensin II) and this would likely counteract their beneficial effects on the pulmonary circulation. An alternative strategy could be to introduce NEP inhibition in combination with an NK1R antagonist, for example. A similar strategy has been trialed for hypertension, using combined NEP/ angiotensin II inhibition with promising results (Cuculi and Erne, 2011).

Another exciting avenue worth pursuing is the interaction of neuropeptides with ET-1. CGRP inhibits the interaction of ET-1 with ET_A_ on vascular smooth muscle cells. This can decrease smooth muscle contraction and is reversible with CGRP antagonists (De Mey and Vanhoutte, 2014). This suggests that intact CGRP may be important in preventing ET-1 activity. Upregulation of CGRP may act as an endogenous ERA inhibitor and attenuate ET-1 over-activity in PAH. There are also emerging findings that substance P may directly interact with ET-1. In non-cardiovascular (melanocyte) cells, substance P can stimulate ET-1 (Park et al., 2015). An NK1R antagonist was able to prevent an increase in ET-1 expression seen in spontaneously hypertensive rats, which was associated with reduced cardiac fibrosis (Dehlin et al., 2013). If the relationship between substance P and ET-1 is proven, this could also have important implications for PAH treatment, given that ET-1 is already a prime target for licensed therapies. Taken together, it would seem that selectivity in regulating neuropeptide release/ activity is of prime importance. Most of the *in vivo* studies in this area have investigated broad-spectrum C-fiber depletion. This is unlikely to give meaningful information on the mechanisms at play in PH. Many of these studies were conducted years ago, prior to the advancements in the efficiency of genome manipulation. It is worth revisiting these neuropeptides in PH. The use of elegant and well-designed studies, utilizing advanced technology, should fully interrogate the vascular interactions of substance P and CGRP in the pulmonary circulation.

## Summary

Pulmonary arterial hypertension is a fatal disease that afflicts people of any age and causes substantial reduction in quality of life. It tends to be more prevalent in young people, particularly women. While treatments developed in the past few decades improved the prognosis for PAH patients, mortality rates still remain high. Current treatment methods are primarily centered on enhancing pulmonary vasodilation. They are not effective at reducing mortality. There is great potential to develop new treatments that target both cardiopulmonary re-modeling and PVR. Altered release of neuropeptides such as substance P and CGRP have been implicated in the pathophysiology of PAH. Selective control of the balance on these neuropeptides in the pulmonary circulation is a promising approach to combating this fatal disease.

## Author Contributions

CL and KB created the figures. CL, SM, and KB researched the literature, prepared the table, and drafted the manuscript.

## Conflict of Interest Statement

The authors declare that the research was conducted in the absence of any commercial or financial relationships that could be construed as a potential conflict of interest.
